# Sequential tumor and dual immune targeted immunotherapy: anti-lymphoma activity of Rituximab with 4-1bb stimulation and PD-1 blockade

**DOI:** 10.1186/2051-1426-2-S3-P106

**Published:** 2014-11-06

**Authors:** Antonia MS Mueller, Jonathan Hebb, Idit Sagiv-Barfi, Aurelien Marabelle, Roch Houot, Amanda Rajapaksa, Debra K Czerwinski, Serena Chang, Cariad Chester, Mohith Sadaram, Erin Waller, Ronald Levy, Holbrook Kohrt

**Affiliations:** 1 University Hospital Zurich, Zurich, Switzerland; 2 Stanford University, Stanford, CA, USA; 3 Centre de Recherche en Cancérologie de Lyon, Université de Lyon, Lyon, France; 4 Service d'Hématologie Clinique, Centre Hospitalier Universitaire de Rennes, Rennes, France

## 

To select maximally-efficacious, minimally-toxic regimens of combination tumor- and immune-targeted therapy, preclinical testing in immune-competent models is required. We previously demonstrated 4-1bb(CD137) stimulation augmented the innate immune response mediated by CD137^+^activated natural killers (NK) cells and the subsequent CD8^+ ^T cell adaptive immune response when administered after tumor-targeting, ADCC-competent, mAbs targeting CD20, HER2, and EGFR. As four ongoing clinical trials (NCT01471210, NCT01775631, NCT02110082, NCT01307267) investigate this strategy, the CD8^+ ^T cell response stimulated by 4-1bb was determined pre-clinically to be further augmented by PD-1/PD-L1 blockade, and a clinical trial is planned (NCT02179918). Taken together, we hypothesized, sequential tumor-targeting with anti-CD20 mAb, Rituximab, followed by dual immune-targeting with anti-CD137 agonism and PD-1/PD-L1 blockade would be efficacious and tolerable due to non-overlapping immune mechanisms and toxicity profiles.

Preclinical modeling was performed in a therapeutic, syngenic, A20 lymphoma BALB/c model combining anti-CD20 mAb (IgG2a-18B12, delivered intraperitoneally, i.p. on d5) with agonistic anti-CD137 mAb (IgG2a-2A, i.p. d6) and anti-PD-1 mAb (IgG2a-RMPI-14, i.p. d6) with intratumoral (i.t.) and circulating (c.) immune responses phenotyped by flow cytometry and time of flight mass cytometry (CyTOF). Combination immunotherapy with anti-CD137 and anti-PD1 was superior to either monotherapy without anti-CD20 treatment in a dose-dependent manner (*p*<.001 tumor-growth, *p <*.001 survival), though no mice were cured long-term. When administered following anti-CD20 treatment, combination immunotherapy with anti-CD137 and anti-PD1 was superior to either monotherapy in a sequence-dependent manner (*p*<.001 tumor-growth, and *p <*.001 survival) with all mice cured long-term and protected from re-challenge when anti-CD20 preceded combination immunotherapy. Target expression was dynamic, as exemplified by highest CD137 expression on i.t.Tregs, followed by i.t.CD8^+ ^T cells. Following anti-CD20 mAb treatment, 1) CD137 expression increased 5-10x on i.t.NKs and 2-6x increase on c.NKs; 2) PD-L1 expression increased on A20 tumor and i.t.CD8^+ ^T cells, and minimally on i.t.NKs; and 3) PD-1 expression increased on i.t.Tregs, i.t.CD8^+ ^T cells, and i.t.NKs. Treatment with anti-CD137 agonist and PD-1 blockade 1) increased the ratio of i.t.NKs/Tregs and c.CD8^+ ^T cells/Tregs, and 2) increased i.t. and c.CD8^+ ^T cell tumor-specific IFN-γ secretion and i.t. and c.NK cell degranulation. Laboratory and necropsy studies identified B cell lymphopenia and mild transaminitis which was notably marked with combination immunotherapy.

We conclude that sequential tumor-targeting followed by dual immune-targeting is highly-efficacious with predictable toxicity and should be considered for clinical translation to augment three therapies with only marginal activity as monotherapy in advanced, relapsed/refractory lymphoma.

